# Janus Kinase 1 Is Required for Transcriptional Reprograming of Murine Astrocytes in Response to Endoplasmic Reticulum Stress

**DOI:** 10.3389/fncel.2019.00446

**Published:** 2019-10-11

**Authors:** Savannah G. Sims, Gordon P. Meares

**Affiliations:** ^1^Department of Microbiology, Immunology, and Cell Biology, West Virginia University, Morgantown, WV, United States; ^2^Department of Neuroscience, West Virginia University, Morgantown, WV, United States; ^3^Rockefeller Neuroscience Institute, West Virginia University, Morgantown, WV, United States

**Keywords:** inflammation, astrocytes, unfolded protein response, protein misfolding, neurodegeneration, Janus kinase, RNA seq, cytokine

## Abstract

Neurodegenerative diseases are associated with the accumulation of misfolded proteins in the endoplasmic reticulum (ER), leading to ER stress. To adapt, cells initiate the unfolded protein response (UPR). However, severe or unresolved UPR activation leads to cell death and inflammation. The UPR is initiated, in part, by the *trans-*ER membrane kinase PKR-like ER kinase (PERK). Recent evidence indicates ER stress and inflammation are linked, and we have shown that this involves PERK-dependent signaling via Janus Kinase (JAK) 1. This signaling provokes the production of soluble inflammatory mediators such as interleukin-6 (IL-6) and chemokine C-C motif ligand 2 (CCL2). We, therefore, hypothesized that JAK1 may control widespread transcriptional changes in response to ER stress. Here, using RNA sequencing of primary murine astrocytes, we demonstrate that JAK1 regulates approximately 10% of ER stress-induced gene expression and is required for a subset of PERK-dependent genes. Additionally, ER stress synergizes with tumor necrosis factor-α (TNF-α) to drive inflammatory gene expression in a JAK1-dependent fashion. We identified that JAK1 contributes to activating transcription factor (ATF) 4-dependent gene expression, including expression of the genes growth arrest and DNA damage (GADD) 45α and tribbles (TRIB) 3 that have not previously been associated with JAK signaling. While these genes are JAK1 dependent in response to ER stress, expression of GADD45α and TRIB3 are not induced by the JAK1-activating cytokine, oncostatin M (OSM). Transcriptomic analysis revealed that JAK1 drives distinct transcriptional programs in response to OSM stimulation versus ER stress. Interestingly, JAK1-dependent genes induced by ER stress in an ATF4-dependent mechanism were unaffected by small molecule inhibition of JAK1, suggesting that, in response to UPR activation, JAK1 initiates gene expression using non-canonical mechanisms. Overall, we have identified that JAK1 is a major regulator of ER stress-induced gene expression.

## Introduction

Prevalent diseases including neurodegenerative disorders, cancer, obesity and diabetes are associated with the accumulation of misfolded proteins in the endoplasmic reticulum (ER) (Oakes and Papa, 2015). Under normal conditions, molecular chaperones within the ER fold proteins, an essential step in the maturation of proteins destined for membranes or secretion. Misfolded proteins can result from a multitude of origins including inflammation, reactive oxygen species (ROS), or genetic mutations (Zhang and Kaufman, 2008; Walter and Ron, 2011). Misfolding can ultimately result in loss of protein function and deleterious effects to the cell. In eukaryotes, the ER has an intricate monitoring system to ensure each protein is properly folded before being exported to its ultimate destination. If a protein is misfolded, mechanisms are in place to re-fold or degrade the aberrant polypeptide. However, when misfolded proteins overwhelm these mechanisms, this results in a disruption of homeostasis, referred to as ER-stress and activation of the unfolded protein response (UPR). The UPR is a highly conserved stress response tasked with restoring homeostasis or initiating apoptosis (Hetz, 2012).

The UPR is mediated by three ER transmembrane sensor proteins: inositol requiring enzyme-1 (IRE1), protein kinase R-like ER kinase (PERK), and activating transcription factor (ATF) 6. Under unstressed conditions, the molecular chaperone glucose regulated protein (GRP78) interacts with and maintains each of these proteins in an inactive conformation. When unfolded proteins accumulate, GRP78 is recruited away from these ER transmembrane proteins, promoting oligomerization and conformational changes in PERK and IRE1, which then likely interact with misfolded polypeptides initiating enzymatic activity (Kimata et al., 2007; Gardner and Walter, 2011; Walter and Ron, 2011; Hetz, 2012; Gardner et al., 2013). Once active, PERK phosphorylates eukaryotic initiation factor 2α (eIF2α) to reduce protein translation and alleviate the influx of nascent polypeptides into the ER (Harding et al., 1999). Concomitantly, the UPR promotes the activation and/or expression of transcription factors such as ATF6, X-box binding protein 1 (XBP1) and ATF4 to drive the expression of ER chaperones to restore function (Walter and Ron, 2011). ER stress has been widely studied in neurons because it is often associated with neuronal death in models of neurological diseases. Increasing evidence indicates ER stress also affects astrocytes. Astrocytes are the most populous glial cell and respond to external stimuli by promoting production of inflammatory cytokines (Sofroniew and Vinters, 2010). In previous studies, humanized ApoE4 and amyloid-β drive ER stress and astrocyte dysfunction. α-synuclein and mutant LRRK2, associated with PD, work together to drive ER stress and Ca^2+^ disruption in astrocytes (Lee et al., 2019). Inflammation and expression of the human endogenous retrovirus protein, syncytin-1, promote ER stress in astrocytes in MS (Deslauriers et al., 2011). Consistent with this, our previous work has indicated that neuroinflammation and STAT3 phosphorylation concomitant with ER stress in the MS mouse model of experimental autoimmune encephalomyelitis (EAE) (Meares et al., 2014). Additionally, we have recently shown that ER stress is transmissible between cells of the CNS. We showed that neurons experiencing ER stress can alert neighboring cells, including astrocytes, by inducing an ER stress response in those cells (Sprenkle et al., 2017). Together, these studies suggest that astrocytes are impacted by ER stress in neurological diseases, and may contribute to the associated pathologies.

In addition, the UPR stimulates an inflammatory response to possibly alert neighboring cells to an impending danger and to recruit immune cells (Zhang and Kaufman, 2008; Sprenkle et al., 2017, 2019). However, this inflammation may contribute to the pathology of diseases involving ER stress (Zhang and Kaufman, 2008; Martinon and Glimcher, 2011; Grootjans et al., 2016). The UPR has been linked to primary signaling molecules contributing to inflammation such as nuclear factor κB (NF-κB), the mitogen activated protein kinase (MAPK) c-Jun N-terminal kinase (JNK), and simulates an acute phase response (Grootjans et al., 2016). The UPR has also been shown to promote the production of cytokines and chemokines, including the pleiotropic cytokine, IL-6 (Li et al., 2005; Meares et al., 2014; Keestra-Gounder et al., 2016). Typically, IL-6 exerts its action by binding to its cell membrane receptor and activating a Janus kinase (JAK) and signal transducer and activator of transcription (STAT) cascade to modulate gene expression (O’Shea and Plenge, 2012). We have previously shown a PERK-dependent mechanism of JAK1 activation leading to IL-6 production, uncovering another connection between ER-stress and inflammation (Meares et al., 2014).

The JAK-STAT pathway is an integral signal transduction pathway in modulating inflammatory gene expression and immunological function (Villarino et al., 2015). Loss of function studies have shown that the 4 JAKs (JAK1, JAK2, JAK3, and Tyk2) and 7 STATs (STAT1, STAT2, STAT3, STAT4, STAT5a, STAT5b, and STAT6) are essential for lymphoid development, T and B cell development, erythropoiesis, defense against viral, and bacterial infections, as well as neural function (Igaz et al., 2001; Nicolas et al., 2013). While the majority of effects elicited by JAK activation are attributed to the activation of STAT proteins, JAKs also integrate with other signaling pathways including phosphatidylinositol 3-kinase (PI3K) signaling and the MAPK pathway (Eulenfeld et al., 2012). Furthermore, cell stressors such as hypoxia, reactive oxygen/nitrogen species, and ER stress activate JAK signaling through receptor-dependent and independent mechanisms (Guzik et al., 2003; Dudley et al., 2004, 2005; Meares et al., 2014).

It is well established that JAK1 is required for responsiveness to interferons, the IL-6 family of cytokines and IL-2, among others, as well as various forms of cell damage. Considering the integral relationship between ER stress and inflammation, we hypothesized that JAK1 may also be a critical signaling node controlling transcriptional changes in response to ER stress. Consistent with this hypothesis, we have identified that JAK1 regulates approximately 10% of the genes induced by ER stress. In addition to its traditional role downstream of cytokine receptors, JAK1 modulates expression of a distinct subset of genes in response to ER stress.

## Materials and Methods

### Mice and Primary Cell Preparations

C57Bl/6, PERK floxed and CAGG-CreE^TM^ mice were purchased from The Jackson Laboratory and bred and housed in the animal facility at West Virginia University under the care of the animal resources program. Primary murine astrocytes were prepared as previously described (Meares et al., 2013). Astrocytes were cultured in Dulbecco’s modified eagle medium (DMEM; Gibco) with 10% fetal bovine serum (FBS; Atlanta Biologicals), 16 mM 4-(2-Hydroxyethyl) piperazine-1-ethanesulfonic acid, N-(2-Hydroxyethyl) piperazine-N′-(2-ethanesulfonic acid) (HEPES; Gibco), 1X non-essential amino acids (Corning), 2 mM L-Glutamine, 100 units/ml penicillin, 100 μg/ml streptomycin (Gibco), and 50 μg/ml gentamicin (Lonza). Astrocytes were separated from microglia by shaking at 200 RPM for 1.5 h. Cells were then trypsinized (0.05%, Gibco) for 5 min at 37°C, collected in media and centrifuged for 5 min at 300*g*. Cells were then seeded into multi-well plates and stimulated after 48–72 h.

### Antibodies and Reagents

Primary antibodies used were: Anti JAK1 (3344), JAK2 (3230), P-eIF2α (3398), eIF2α (5324), P-STAT3 (9145), STAT3 (12640), Lysine-specific histone demethylase 1 (LSD1) (2184), ATF4 (11815) from Cell Signaling; Glyceraldehyde 3-phosphate dehydrogenase (GAPDH) (MAB374) from Millipore; JAK1 (610231) from BD Transduction Laboratories; transferrin receptor (TfR) H68.4 (13-6800) from Thermo Fisher; and CCAAT-enhancer-binding protein homologous protein (CHOP) (sc-7351) from Santa Cruz. Cytokines used were: oncostatin (OSM) M and tumor necrosis factor (TNF) – α from R&D systems. Thapsigargin and tunicamycin used were from EMD Millipore and AZD1480 was supplied from Santa Cruz Biotechnology. Puromycin was supplied from Fisher Scientific.

### Immunoblotting

Cells were washed twice with phosphate buffered saline (PBS) and lysed with lysis buffer (20 mM 2-Amino-2-(hydroxymethyl)-1,3-propanediol (Tris), pH 7.5; 150 mM NaCl; 2 mM Ethylenediaminetetraacetic acid (EDTA); 2 mM Ethylene-bis (oxyethylenenitrilo) tetraacetic acid (EGTA); 0.5% Non-idet P-40 (NP-40) containing 1X phosphatase and protease inhibitor cocktail (Pierce) as previously described (Meares et al., 2004). Protein concentrations were determined using the bicinchoninic acid assay (Pierce). Equal amounts of protein from each sample were solubilized in Laemmli sample buffer and heated for 5 min at 95°C. Proteins were separated by SDS-polyacrylamide gel electrophoresis, transferred to nitrocellulose, and the membranes were blocked in 5% milk/tris buffered saline with tween-20 (TBST), followed by an overnight incubation at 4°C with primary Ab diluted in 5% bovine serum albumin (BSA) or milk in TBST, according to the manufacturer’s recommendation. Horseradish peroxidase-conjugated donkey anti-rabbit or donkey anti-mouse (1:4000 dilution) secondary Ab (Jackson Immuno Research) were incubated for 1 h at room temperature, followed by detection with enhanced chemiluminescence. Membranes were imaged digitally using a ChemiDoc Touch (Biorad). Immunoblot images were analyzed using ImageLab software (BioRad). When applicable, quantification of immunoblot images were quantified by obtaining volumetric measurements in ImageLab.

### qRT-PCR

RNA was isolated using 1 ml of TRIzol (Sigma-Aldrich) according to the manufacturer’s instructions. RNA was quantified using a NanoDrop (NanoDrop Technologies), and 1 μg of RNA was used for cDNA synthesis using Moloney Murine Leukemia Virus reverse transcriptase (Promega). The cDNA was analyzed by quantitative PCR performed using probe-based gene expression assays (IDT or Themo Fisher) in a Stratagene MX3005P or Applied Biosystems Quant Studio 3. Reactions were carried out in 20 μL and analyzed using the ΔΔCt method.

### Protein Translation

Protein synthesis was estimated by measuring puromycin incorporation using a modified method based on (Schmidt et al., 2009). Briefly, cells were incubated with puromycin (5 μg/ml) for 5 min followed by washing in cold PBS and lysed with lysis buffer. One microgram of protein was spotted in duplicate or triplicate on nitrocellulose and allowed to dry. The membrane was then immunoblotted (dot blot) using an anti-puromycin antibody (Millipore) at 1:5000 dilution in 5% milk/TBST. Dots were quantified using ImageLab software (Biorad).

### RNA Sequencing and Bioinformatics

RNA was quantified by Qubit fluorometer. RNA quality was assessed by Bioanalyzer Nano chip. All RIN values were greater than 8. Libraries were built using 750 ng RNA and KAPA stranded mRNA kit as per manufacturers protocol. The libraries were then quantified with the Qubit and run on the Bioanalyzer using a High Sensitivity DNA chip to determine average size. They were then pooled at an equimolar ratio and sequenced (paired end (PE) 100 bp) on the HiSeq 2500 at Marshall University. RNA seq was also performed externally by Genewiz. Analysis was performed using CLC Biomedical Genomics Workbench and Ingenuity Pathway Analysis (Qiagen). Non-coding or non-annotated genes were not included in analysis. Gene ontology was analyzed using ShinyGO v0.60 http://bioinformatics.sdstate.edu/go/(Ge and Jung, 2018). Full data sets are available at NCBI Sequence Read Archive (SRA) # SRP129889.

### Immunoprecipitation

Protein lysates were collected in lysis buffer. Anti-rabbit Dynabeads (15 μl per sample, Invitrogen) were coated with 1 μg of α-ATF4 antibody overnight. Beads with the α-ATF4 antibody were washed with PBS with 0.1% BSA 3 times. Protein (750 μg) was then incubated with the Dynabeads for 3 h and washed two times with 0.5% NP-40 lysis buffer and two times with PBS with 0.1% BSA. Protein was eluted by incubating the Dynabeads in 1X Laemelli Buffer at 95°C for 5 min.

### Cellular Fractionation

Nuclear and cytoplasmic fractions were obtained by collecting cells in 0.05% NP-40 buffer (10 mM Tris pH 7.4, 10 mM NaCl, 3 mM MgCl_2_, 1 mM EGTA, 0.05% NP-40 with 1X protease and phosphatase inhibitors) and centrifuged at 2700 × *g* for 10 min at 4°C. Supernatants were collected and centrifuged at 17,000 × *g* for 15 min at 4°C to obtain cytoplasmic fractions. The pellet containing nuclei was washed twice in 200 μl of wash buffer (5 mM HEPES, pH 7.4, 3 mM MgCl_2_, 1 mM EGTA, 250 mM sucrose, 0.1% BSA, with 1X protease and phosphatase inhibitors). The pellet was then resuspended in wash buffer and layered on top of 1 ml of 1 M sucrose (with protease and phosphatase inhibitors), and centrifuged at 2700 × *g* for 10 min at 4°C. The nuclear pellet was washed in the 0.05% NP-40 lysis buffer. The nuclear proteins were extracted by resuspending the pellet in nuclear extraction buffer (20 mM HEPES pH 7.4, 1.5 mM MgCl_2_, 0.2 mM EDTA, 10 mM β-glycerophosphate, 300 mM NaCl with 1X protease and phosphatase inhibitor) and incubating on ice for 30 min. The nuclear fractions were subsequently centrifuged at 17,000 *g* for 15 min at 4°C. The supernatant was saved as nuclear extract.

### siRNA Transfections

Primary astrocytes were transfected with the indicated small interfering (si) RNA (50 pmols per 35 mm well) using Lipofectamine RNAiMAX (Life Technologies) according to the manufacturer’s protocol. Cells were used for experiments 48–72 h after transfection. The siRNAs used in this study include Control (non-targeting) siRNA, JAK1 siRNA #1 (sequence: GCUCCGAACCGAAUCAUCA), JAK1 siRNA #2 (sequence: CACUGAUUGUCCACAAUAUTT), JAK2 siRNA (sequence GGACUAUAUGUGCUACGAUTT), ATF4 siRNA #1 (sequence: GCUGCUUACAUUACUCUAATT), ATF4 siRNA #2 (sequence: GCCUAGGUCUCUUAGAUGATT).

### ELISA

Culture supernatants (100 μL, undiluted) were collected and assayed by ELISA for murine IL-6 (Biolegend) according to the manufacturer’s protocol.

### Statistics

Data are the means of at least three independent experiments. Significance, indicated by ^∗^where *p* < 0.05, was determined by one-way analysis of variance (ANOVA) with *post hoc* analysis or by Student’s *t*-test. RNA-seq significance was determined using Empirical Analysis of Differential Gene Expression (EDGE) test (Robinson and Smyth, 2008; Robinson et al., 2010).

## Results

**FIGURE 1:**
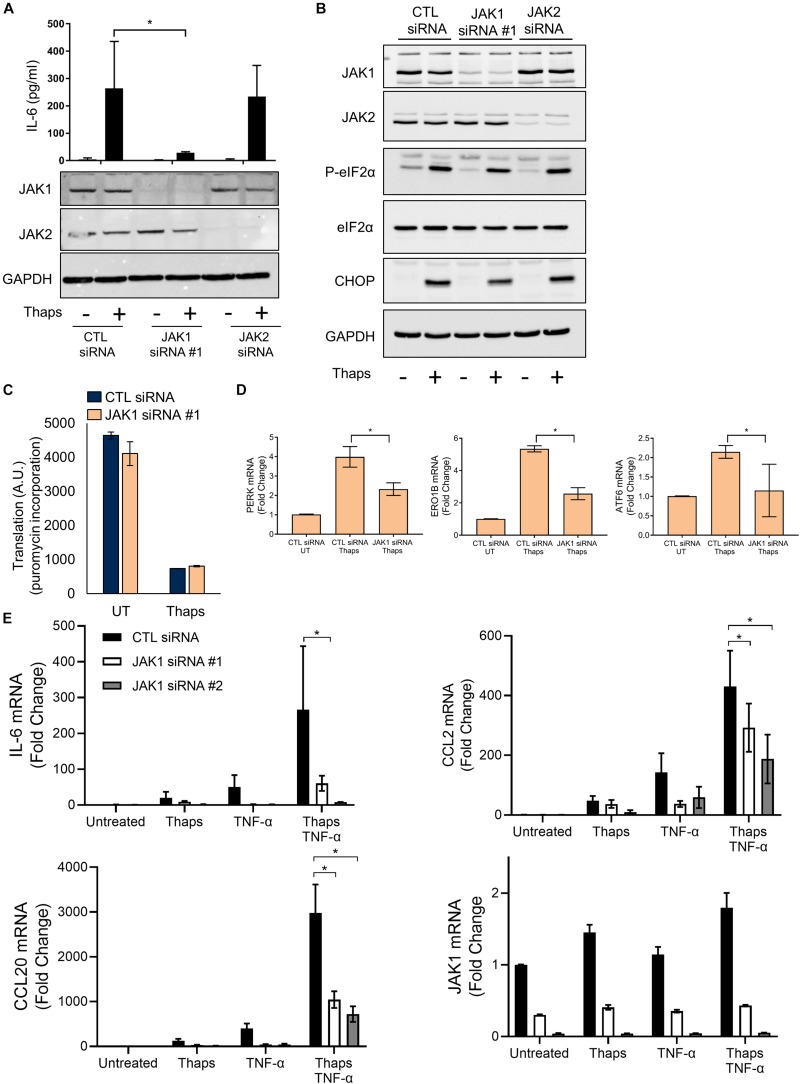
JAK1 is required to drive ER stress-induced IL-6 expression and does not affect canonical PERK signaling. **(A)** Primary astrocytes were transfected with control (CTL), JAK1, or JAK2 siRNA for 48 h and then treated with thapsigargin (thaps) (1 μM) for 24 h and analyzed by immunoblot and ELISA. **(B)** Astrocytes were transfected as in **(A)** followed by thaps (1 μM) treatment for 4 h and analyzed by immunoblot. **(C)** Astrocytes were transfected with CTL or JAK1 siRNA and treated with thaps (1 μM) for 90 min. In the last 5 min of treatment, puromycin (5 μg/ml) was added to cultures. Cell lysates were analyzed by dot blot using anti-puromycin antibody and quantified. UT = Untreated. **(D)** Astrocytes were transfected as in **(A)**, treated with thaps (1 μM) for 4 h then analyzed by RT-qPCR. UT = Untreated. **(E)** Primary astrocytes were transfected with one of two distinct JAK1 siRNAs and treated with thaps (1 μM), TNF-α (5 ng/ml), or both thaps and TNF-α for 4 h and analyzed by RT-qPCR. *N* = 3. ^∗^*p* ≤ 0.05. Data are represented as means ± standard deviation.

In this study, we have used primary murine astrocytes as a model to study the role of JAK1 in the ER stress response. Astrocytes are resistant to the cytotoxic effects of prototypical ER stress inducing agents but respond with a robust UPR and inflammatory response (Meares et al., 2014), making them ideal to study signaling and gene expression without overt cell death. We have previously shown that ER stress-induced IL-6 expression requires PERK and JAK1 in astrocytes (Meares et al., 2014). To extend these findings, we tested if JAK2 could also regulate IL-6. We focused on testing JAK1 and JAK2 because other JAKs (JAK3 and Tyk2) are lowly expressed in astrocytes (Zhang et al., 2014). As shown in Figure 1A, ER stress induced by thapsigargin (thaps) drives production of IL-6 and siRNA-mediated knockdown of JAK1 abrogated ER stress-induced IL-6 production, while JAK2 knockdown had no effect in comparison to the control (non-targeting) siRNA. Other JAK proteins, Tyk2, and JAK3, are lowly expressed in astrocytes, suggesting they do not play an appreciable role in ER stress-induced signaling (Zhang et al., 2014). Next, to understand how JAK1 affects ER stress-induced signaling, we tested if JAK1 or JAK2 could modulate canonical PERK signaling. JAK1 or JAK2 was knocked down in astrocytes, and the cells were exposed to thaps for 4 h to induce ER stress. Knockdown of JAK1 and JAK2 was highly effective and selective, but this had no significant impact on PERK-dependent eIF2α phosphorylation or CHOP expression (Figure 1B). Because JAK2 had no effect on driving thaps-induced IL-6 production and did not affect the UPR signaling pathway, we chose to focus our studies solely on JAK1. Moreover, eIF2α phosphorylation leads to translational repression, and this is also unaffected by JAK1 knockdown (Figure 1C). JAK/STAT signaling drives transcriptional changes, therefore, we tested if JAK1 could regulate expression of UPR signal transducers and ER chaperones. As shown in Figure 1D, ER stress increased the expression of PERK, ATF6, and the oxidoreductase ER oxidoreductin-like beta (Ero1lb) dependent on JAK1. These data indicate that JAK1 activation is dispensable for PERK dependent signaling that leads to translational repression, and imply a unique role for JAK1 in the regulation of the ER stress response. UPR signaling has also been shown to augment already ongoing inflammatory responses, including NF-κB signaling (Hotamisligil, 2010; Kitamura, 2011; Tam et al., 2012). To determine if JAK1 plays a role in mediating synergy between inflammatory and UPR signaling, we treated astrocytes with thaps and the proinflammatory cytokine, TNF – α, which engages NF-κB signaling (Schütze et al., 1995). Here, we also chose a second JAK1-targeting siRNA to corroborate our findings that this response is specific to JAK1. We show that inflammatory gene expression (IL-6, CCL2, and CCL20) responds in a synergistic manner to ER stress and TNF-α. Further, this synergy is JAK1 dependent, highlighting the role of ER stress in influencing astrocyte-dependent inflammatory responses (Figure 1E). We have defined synergy here, as ER stress having a more than additive effect on the TNF-α stimulated inflammatory response. Overall, these data suggest that JAK1 drives transcriptional regulation during UPR activation in astrocytes.

**FIGURE 2:**
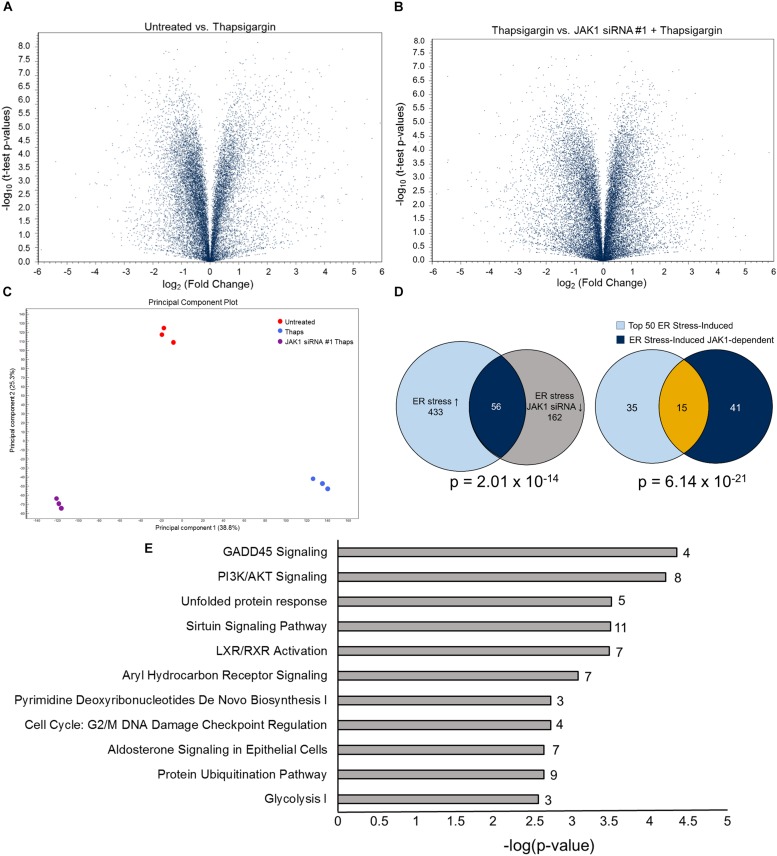
JAK1 regulates approximately 10% of ER stress-induced gene expression. **(A)** Primary astrocytes were transfected with control (CTL) or JAK1 siRNA for 48 h followed by treatment with thaps (1 μM) for 4 h and analyzed by RNA-seq. Volcano plots represent fold change and *p*-values for approximately 25,000 expressed genes. **(B)** Results were analyzed as in **(A)** between thaps-treated samples without or with JAK1 knockdown. **(C)** Principle Component Analysis (PCA) of RNA-seq treatment groups. **(D)** Venn diagram showing overlap of genes upregulated in response to thaps treatment and downregulated by JAK1 knockdown (left). Venn diagram of top 50 ER stress-induced genes overlapping with JAK1-dependent genes (right). **(E)** Functional classification of genes identified to be JAK1-dependent (Venn diagram overlap – 56 genes) in response to ER stress using Ingenuity Pathway Analysis. The number of JAK1-regulated genes in each pathway is indicated adjacent to each bar.

These findings, with our previous work showing that JAK1 regulates IL-6, CCL2 and CCL20 expression, led us to hypothesize that JAK1 has an important role in regulating the transcriptional response to ER stress (Meares et al., 2014). To test this globally, we used RNA sequencing (RNA-seq). Astrocytes were transfected with control or JAK1 siRNA followed by treatment with thaps for 4 h. Global changes in the transcriptome were then analyzed by RNA-seq. As shown in the volcano plot in Figure 2A, ER stress induces transcriptional reprograming including upregulation of the prototypical UPR genes CHOP (ddit3), ATF4 and XBP1 (Supplementary Figure S1). When JAK1 was knocked down in ER stressed cells, this appeared to change the expression of many genes when compared to thaps alone based on *t*-test *p*-values (Figure 2B). These data suggested that both ER stress and JAK1 had a significant impact on the overall gene expression profile. To test this, we used principal component analysis (PCA) which revealed that each of the treatment groups had a unique expression profile, indicating that JAK1 regulates the overall response to ER stress (Figure 2C). We next investigated the global impact of JAK1 on ER stress-induced gene expression using stringent statistical analysis. We identified all of the genes significantly (EDGE test *p* < 0.05) upregulated by 1.5-fold or greater in response to ER stress. We then identified all the ER stress-induced genes that are JAK1 dependent. These were genes significantly upregulated by ER stress and significantly reduced by 1.5-fold or greater by JAK1 knockdown. Overall, more than 450 genes were increased by ER stress and approximately 10% of these genes were regulated by JAK1 (Figure 2D). These data indicate that JAK1 has a significant [*p* = 2.01 × 10^–14^ by hypergeometric probability (Fury et al., 2006)] and unexpectedly large role in the regulation of ER stress-induced gene expression. To examine the most strongly induced genes, we identified the top 50 ER stress-induced genes (Supplementary Figure S1). This list included well-established genes known to be robustly induced by ER stress including tribbles 3 (TRIB3), CHOP, and ATF3 (Han et al., 2013). We then compared this gene set to the ER stress induced genes that are JAK1-dependent (Supplementary Figure S1). This identified CCL20, which we previously identified as JAK1-dependent as well as many genes not previously associated with JAK1 signaling. By comparing these two analyses, we identified that 15 (30%) of the top 50 ER stress-induced genes are JAK1 dependent (Figure 2D). This is a highly significant overlap (*p* = 6.14 × 10^–21^ by hypergeometric probability). These included adrenomedullin 2 (Adm2), CCL20, Prostaglandin-endoperoxide synthase 2 (Ptgs2), Nuclear Protein 1 (Nupr1) and Regulator of G Protein Signaling (RGS) 16 among others, which have previously been shown to be induced by ER stress (Hidvegi et al., 2007; Cho et al., 2011; Huang et al., 2011; Meares et al., 2014; Kovaleva et al., 2016). To identify the general pathways regulated by JAK1, we used Ingenuity Pathway Analysis (IPA). As shown in Figure 2E, growth arrest and DNA damage (GADD) 45α signaling and other stress-responsive pathways, including the UPR, were significantly regulated by JAK1. These data indicate that JAK1 has a central role in the regulation of transcriptional reprograming induced by ER stress.

**FIGURE 3:**
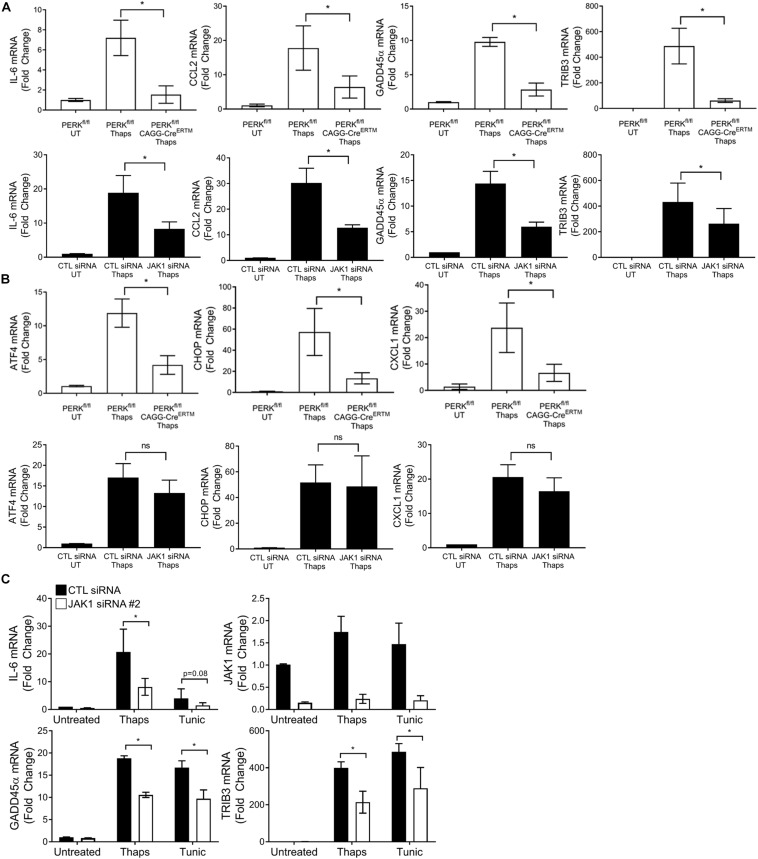
JAK1 is required for full engagement of PERK-dependent gene expression. **(A,B)** Astrocytes were isolated from PERK^fl/fl^ mice without or with tamoxifen-inducible cre (CAGG-CreER^TM^). Cells were treated with tamoxifen for 48 h to delete PERK followed by thaps (1 μM) treatment for 4 h and RT-qPCR analysis. Astrocytes were transfected with CTL or JAK1 siRNA #1 for 48 h, treated with thaps (1 μM) for 4 h, and analyzed by RT-qPCR. **(C)** Astrocytes were transfected with a second JAK1 siRNA, and ER stress was induced by treating with thaps (1 μM) or tunicamycin (tunic) (5 μM) for 4 h, followed by RT-qPCR analysis. Data are represented as means ± standard deviation. *N* = 3. ^∗^*p* ≤ 0.05.

We have previously shown PERK-dependent activation of JAK1 (Meares et al., 2014). Therefore, we expected that JAK1 would be important for PERK-dependent transcriptional responses. To test this, we selected several genes including IL-6 and CCL2 that we know to be PERK and JAK1 dependent. We also selected, based on the RNA-seq data, the DNA damage induced protein GADD45α and the pseudokinase TRIB3. As shown in Figure 3A, ER stress induces the expression of IL-6, CCL2, GADD45α, and TRIB3. Genetic deletion of PERK significantly reduced ER stress induced expression of each of these genes, indicating they are PERK dependent. JAK1 knockdown also significantly suppressed each of these genes, indicating they are JAK1 dependent (Figure 3A). Importantly, not all PERK-dependent gene expression relies on JAK1. As shown in Figure 3B, ER stress-induced expression of ATF4, CHOP and the chemokine C-X-C motif ligand 1 (CXCL1) are PERK dependent but are unaffected by JAK1 knockdown. These data demonstrate that JAK1 is essential for full engagement of PERK-dependent gene expression in response to ER stress. These data suggest, for the first time, that GADD45α and TRIB3 are JAK1 dependent. GADD45α and TRIB3 are known to be induced by ER stress, however, have not been previously associated with JAK-STAT signaling. To confirm that ER stress upregulates GADD45α and TRIB3 expression in a JAK1-dependent manner, we utilized a different ER stress-inducing agent (tunicamycin) and a second distinct JAK1 siRNA. In Figure 3C, we corroborated that JAK1 knockdown reduces ER stress-induced expression of IL-6, GADD45α, and TRIB3, providing further evidence that these genes are JAK1-dependent.

**FIGURE 4:**
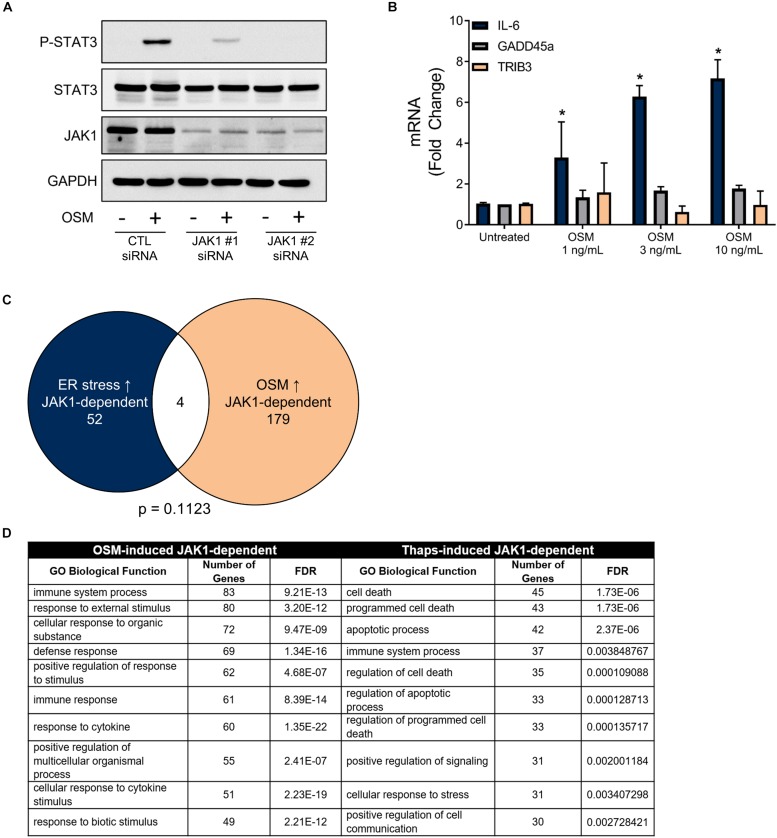
ER stress induces a unique JAK1-dependent gene expression profile that is distinct from OSM-induced JAK1-dependent gene expression. **(A)** Primary astrocytes were transfected with Control (CTL) or one of two distinct JAK1 siRNAs for 48 h and stimulated with OSM for 30 min followed by immunoblotting. **(B)** Astrocytes were stimulated with OSM at the indicated concentrations for 4 h. Gene expression was measured by RT-qPCR. Data are represented as means ± standard deviation. *N* = 3. ^∗^*p* ≤ 0.05. **(C)** Astrocytes were transfected with CTL or JAK1 siRNA#2 and treated with OSM (2.5 ng/ml) for 4 h. Gene expression was then measured by RNAseq to identify the JAK1-dependent genes. Venn diagram of JAK1 dependent genes in response to ER stress or OSM. **(D)** Gene ontology analysis of the genes represented in **(C)**.

Next, we tested if a JAK1-activating cytokine could also drive GADD45 and TRIB3 expression. We used the IL-6 family cytokine, oncostatin M (OSM), which signals through JAK1-STAT3-dependent mechanisms in astrocytes (Zhang et al., 2014). JAK1 siRNA knockdown in astrocytes led to an abrogation of OSM-mediated phosphorylation of STAT3, confirming the requirement of JAK1 (Figure 4A). Stimulation of astrocytes with OSM induced a concentration-dependent increase of IL-6, as expected. However, OSM had no effect on GADD45α or TRIB3 expression (Figure 4B). Next, we took a transcriptome-wide approach to compare the set of JAK1-dependent genes in response to OSM versus ER stress (Figure 2B). Here, we used RNA-seq to identify significantly induced (EDGE test *p*-value < 0.05) genes by OSM. These genes had a fold change of 1.5 or greater when compared to untreated samples. Next, we identified genes that were significantly downregulated (EDGE test *p*-value < 0.05, fold change < -1.5) with JAK1 knockdown. These criteria allowed us to identify the 183 OSM-induced JAK1-dependent genes. We then compared the genes that are induced by ER stress and OSM in a JAK1-dependent fashion. This revealed strikingly disparate gene expression profiles, with only four genes in common (Figure 4C). The genes that are JAK1 dependent in response to both OSM and ER stress are pentraxin 3 (Ptx3), nuclear protein 1 (Nupr1), Regulator of G Protein Signaling (Rgs) 16, and chemokine (C-C motif) ligand (CCL) 7. These data suggest that, in astrocytes, cytokines and ER stress induce distinct JAK1-dependent gene expression changes. Next, we performed gene ontology analysis which assigns genes to groups based on their molecular and functional characteristics previously defined in the literature. Gene ontology showed JAK1 regulates gene expression corresponding to different biological process depending on the stimulus (OSM or ER stress) (Figure 4D). OSM-induced JAK1 dependent genes generally induce immune and inflammatory related genes. However, ER stress-induced JAK1 dependent genes are related to cell death and apoptosis. This highlights that ER stress engages JAK1 to control a distinct transcriptional profile in comparison to the well-established role of JAK1 downstream of cytokine receptors that we have modeled using OSM stimulation.

**FIGURE 5:**
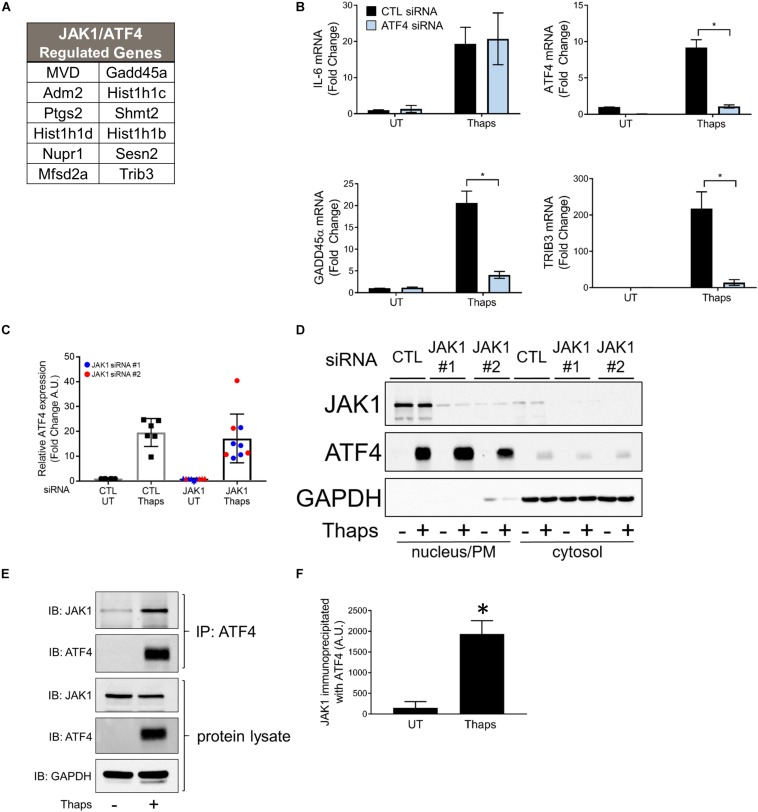
JAK1 and ATF4 cooperatively regulate a subset of ER stress-induced genes. **(A)** List of genes that are both JAK1 and ATF4 dependent in response to ER stress as determined by RNA-seq and reported by others. **(B)** Astrocytes were transfected with control (CTL) or ATF4-targeting siRNA for 48 h and treated with thapsigargin for 4 h. Indicated gene expression was analyzed by RT-qPCR. UT = untreated. **(C)** Astrocytes were transfected with CLT or one of two JAK1 siRNAs and immunoblotted for ATF4. Immunoblots were quantified and normalized to GAPDH expression. **(D)** Astrocytes were transfected with CTL or JAK1 siRNA for 48 h and then treated with thapsigargin for 4 h. Cytosolic and nuclear fractions were isolated and analyzed by immunoblot. **(E)** Primary astrocytes were treated with thaps (1 μM) for 4 h. Protein lysates reserved or immunoprecipiated with α-ATF4 antibody before immunoblotting. **(F)** Quantification of JAK1 co-immunoprecipiated with ATF4 as shown in the top panel of (E). Data are represented as means ± standard deviation. *N* = 3. ^∗^*p* ≤ 0.05.

Previous work has shown that GADD45α and TRIB3 are ATF4 dependent (Jiang et al., 2007; Liew et al., 2010). ATF4 is a transcription factor known to be induced by cell stress, including ER stress. Expression of ATF4 is initiated downstream of PERK activation, requiring the phosphorylation of eIF2α (Wek et al., 2006). Further, we determined that many genes are regulated by both JAK1 and ATF4 in response to ER stress and identified that 12 (out of 56) ER stress-induced JAK1-dependent genes have been previously reported as ATF4-dependent (Passe et al., 2006; Lange et al., 2008; Oyadomari et al., 2008; Cho et al., 2011; Brüning et al., 2013; DeNicola et al., 2015; Kovaleva et al., 2016) (Figure 5A). To confirm that GADD45α and TRIB3 are ATF4-dependent, we used siRNA to knockdown ATF4. As shown in Figure 5B, ATF4 knockdown abrogated ER stress-induced GADD45α and TRIB3, but failed to reduce expression of IL-6, consistent with our previous work (Guthrie et al., 2016). These findings were confirmed using a second, distinct siRNA targeting ATF4 (Supplementary Figure S2). This suggests that JAK1 and ATF4 may cooperatively regulate gene expression. To determine if JAK1 regulated protein expression of ATF4, we quantified ATF4 immunoblots of thaps-treated astrocytes with or without JAK1 knockdown. JAK1 knockdown had no significant effect on ATF4 protein levels in response to ER stress (Figure 5C). Previously, proteins related to JAK1 and ATF4, JAK2, and CREB, respectively, have been shown to interact and translocate to the nucleus (Lefrancois-Martinez et al., 2011). Although JAK1 knockdown does not affect ATF4 expression, we next tested if JAK1 expression is required for nuclear translocation of ATF4. We found that ATF4 is expressed in the nucleus in response to thaps treatment independent of JAK1 expression, indicating that JAK1 does not influence the expression or nuclear translocation of ATF4 (Figure 5D). Although JAK1 appeared in the nuclear fraction under these conditions, analyzing cytosolic, nuclear, and plasma membrane markers indicated that the nuclear fraction also contained plasma membrane (detected by the presence of transferrin receptor, Supplementary Figure S3). JAK1 is largely associated with the plasma membrane (Behrmann et al., 2004). To test if JAK1 and ATF4 physically interact, protein lysates from thaps-treated astrocytes were immunoprecipitated using anti-ATF4 antibody and immunoblotted for ATF4 and JAK1. Here, we found that JAK1 coimmunoprecipitates with ATF4, suggesting a physical interaction between these two molecules (Figures 5E,F). Altogether, these data suggest that JAK1 and ATF4 cooperatively regulate ER stress-induced gene expressions.

**FIGURE 6:**
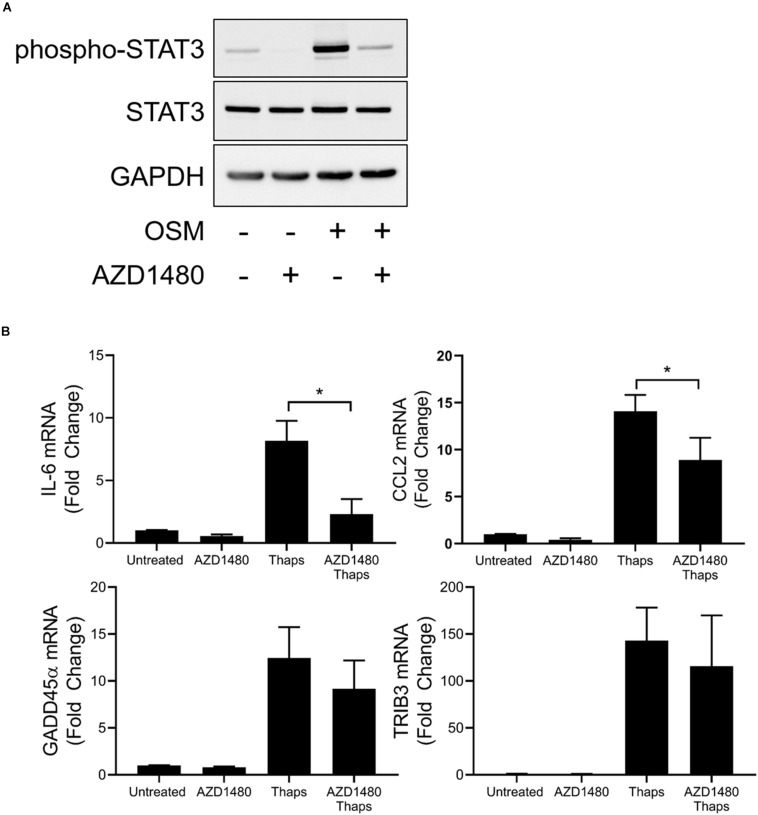
A subset of ER stress-induced JAK1-dependent signaling is insensitive to kinase inhibition of JAK1. **(A)** Astrocytes were pretreated with JAK1/2 kinase inhibitor AZD1480 (1 μM) for 1 h before 0.5 h treatment with oncostatin M (OSM). Protein lysates were collected and analyzed by immunoblot. **(B)** Astrocytes were pretreated with AZD1480 for 1 h and then treated with thaps for 4 h. Gene expression was analyzed by RT-qPCR. Data are represented as means ± standard deviation. *N* = 3. ^∗^*p* < 0.05.

The role of JAK1 to direct transcription factor activity in response to cytokines and growth factors is well established to rely on tyrosine phosphorylation. JAKs phosphorylate STATs to induce dimerization and translocation to the nucleus to initiate gene expression (Schwartz et al., 2017). Because we have shown that JAK1 and ATF4 regulate common genes and coimmunoprecipitate, we hypothesize that ATF4 could be an alternative transcription factor that JAK1 can phosphorylate to alter activity in response to UPR activation. To determine if the kinase activity of JAK1 is necessary to promote ER stress-induced ATF4-dependent gene expression, astrocytes were treated with the JAK1/2 kinase inhibitor AZD1480 and thaps. As shown in Figure 6A, AZD1480 effectively abrogates OSM-induced phosphorylation of STAT3. Expression of known JAK1/STAT3-dependent genes IL-6 and CCL2 were increased by thaps and kinase inhibition of JAK1 attenuated expression of these genes (Figure 6B). However, GADD45α and TRIB3 were not sensitive to JAK1 kinase inhibition (Figure 6B). These results imply that JAK1 elicits non-canonical signaling in response to ER stress that may not rely on the kinase activity of JAK1. These data suggest that JAK1, through physical interaction, can influence ATF4-dependent gene expression.

## Discussion

**FIGURE 7:**
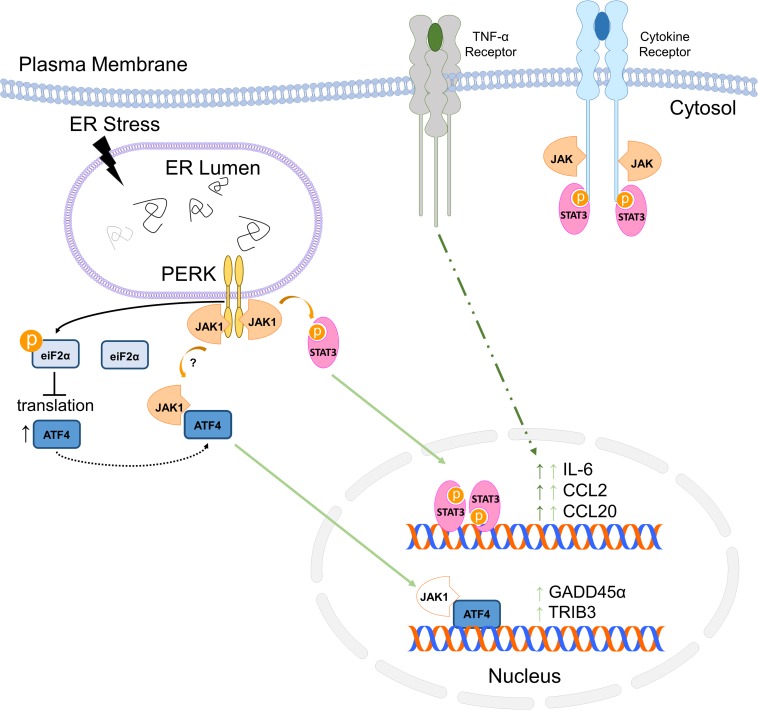
Summary of ER stress-induced JAK1-dependent signaling.

In this study, we have shown that JAK1 controls the expression of an unexpectedly large number of genes in response to ER stress. Many of the genes regulated are associated with inflammation, consistent with the critical and well-established role of JAK1 in immune function (O’Shea and Plenge, 2012; Villarino et al., 2015). We and others have shown that UPR signaling integrates with multiple pathways regulating inflammation (Zhang and Kaufman, 2008; Sprenkle et al., 2017). We have established that the PERK-JAK1 axis drives inflammatory gene expression including IL-6 in murine astrocytes and other cell types (Meares et al., 2014; Guthrie et al., 2016) while IRE1 drives IL-6 through a nucleotide binding oligomerization domain 1/2 (NOD1/2) dependent mechanism in macrophages and in the periphery *in vivo* (Keestra-Gounder et al., 2016). JAK1 also regulated genes involved in the ER stress response including the key signal transducing molecules PERK and ATF6. Further, our previous work has shown that JAK1 interacts with PERK and that PERK is phosphorylated by JAK1. Our current work demonstrates that JAK1 is a critical mediator of PERK-dependent gene expression but does not regulate phosphorylation of eIF2α or subsequent attenuation of protein translation. These data suggest a reciprocal interaction in which PERK drives JAK1 activation, which in turn, drives PERK expression. This work also suggests that PERK-dependent activation of JAK1 and phosphorylation of eIF2α are distinct signaling branches. While PERK appears to initially stimulate independent pathways through JAK1 and eIF2α, we have shown that ER stress-induced IL-6, CCL2, and CCL20 expression require both JAK1 and translational attenuation independent of ATF4 (Guthrie et al., 2016). Further, we have shown that JAK1 mediates synergistic gene expression between ER stress and the proinflammatory cytokine TNF-α. Our findings, here, are summarized in Figure 7. This is consistent with other reports that ER stress is able to augment ongoing inflammatory responses (Liu et al., 2012; Rao et al., 2014; Keestra-Gounder et al., 2016; Reverendo et al., 2019), and for the first time, have shown that JAK1 is integral for this synergy in astrocytes. We have now identified that JAK1 modulates ATF4-dependent gene expression, indicating that JAK1 integrates at multiple points downstream from PERK. In some contexts, ER stress has been reported to inhibit JAK/STAT signaling (Gunduz et al., 2012; Kimura et al., 2012). However, additional studies are needed to determine if JAK1 also drives non-canonical gene expression in those cell types and conditions.

Our current work demonstrates that JAK1 is a critical signaling node in response to ER stress in astrocytes. However, we cannot distinguish if the JAK1-dependent effects are immediate to ER stress signaling or result from basal regulation in the expression of critical signaling molecules. The data indicate that JAK1 can drive stimulus-dependent gene expression programs. We have shown that JAK1 dependent genes in response to cytokine (OSM) stimulation is distinct from ER stress driven JAK1-dependent gene expression. We show this JAK1 promoted the expression of genes such as GADD45α and TRIB3 in response to ER stress but not following cytokine stimulation. It is currently unknown if this differential JAK1-dependent gene expression program is an adaptive response or part of a maladaptive response to ER stress and potentially other pathogenic stimuli. Moreover, pathway analysis implicated JAK1 in the regulation of several stress-activated pathways not investigated in the present study, such as sirtuin signaling, PI3K/AKT signaling, aryl hydrocarbon receptor signaling, and protein ubiquitination pathways (Figure 2E). JAK1 has broad involvement in mediating the biological actions of many cytokines such as the IL-6 family, IL-10 family, and interferons (Müller et al., 1993; Finbloom and Winestock, 1995; Riley et al., 1999; Bousoik and Montazeri Aliabadi, 2018). Additionally, JAK1 inhibitors are under investigation for treatment of cancers and autoimmunity (Schwartz et al., 2017). However, this treatment may promote immunosuppression; upper respiratory tract and urinary tract infections were among the most common side effects reported in psoriasis patients using JAK1 inhibitors (Hsu and Armstrong, 2014). Therefore, a complete understanding of the JAK1-dependent mechanisms induced by both cytokines and cellular stress may provide broad insight into the mechanisms that underlie pathology-associated signaling pathways.

The nature of the novel JAK1 signaling activity is currently unknown but, as we have shown, may involve interaction with the stress-inducible transcription factor ATF4. We have shown that JAK1 and ATF4 regulate many of the same genes in response to ER stress and that JAK1 coimmunoprecipitates with ATF4. However, as suggested in Figure 6, the JAK1-mediated regulation of ATF4 may not involve the well-characterized kinase activity of JAK1. Although we used a kinase inhibitor of JAK1 that also inhibits kinase activity of JAK2, we do not believe that JAK2 plays an appreciable role in regulated ER stress-induced gene expression (Figure 1A). Expanded studies to confirm this kinase-independent interaction between JAK1 and ATF4 are currently underway. Other potential mechanisms include JAK1 nuclear localization (Zhu et al., 2017) and modulation of gene expression or a structural/adaptor function to facilitate key signaling events such as the activation of other transcription factors, like ATF4. JAK1 contains functional domains including a FERM domain and a pseudokinase domain which may mediate important non-catalytic functions of JAK1 (Yamaoka et al., 2004). In models of diffuse large B cell lymphoma, others have recently elucidated that JAK1 has a classical nuclear localization sequence between its FERM and SH2 domains, demonstrating that JAK1 may influence transcriptional changes using various mechanisms that are independent of STAT phosphorylation at the site of cytoplasmic cytokine receptors (Zhu et al., 2017). Further, a non-canonical role for JAK1 has been described in epigenetic modulation of gene expression. JAK1 has been shown to directly phosphorylate the histone protein, H3, to promote STAT-independent gene expression (Rui et al., 2016).

While we have revealed an important and previously unknown role for JAK1 in response to ER stress, there are several caveats. First, this work was completed using a single type of cultured cells (primary astrocytes) and high concentrations of pharmacological agents to induce ER stress. It is unknown from these data if JAK1 has a similarly important role *in vivo* under physiological conditions. These studies are currently underway. Additionally, we focused on measuring gene expression at the mRNA level because of the tools available for whole genome transcriptomics. Considering that most translation is inhibited by ER stress-induced eIF2α phosphorylation (Harding et al., 1999), it is likely that many of the transcripts we have measured are not translated into proteins. Nonetheless, this transcriptional reprograming may be important following resolution of ER stress and resumption of translation. Overall, our data indicate that JAK1 is a central mediator of transcriptional reprograming during ER stress.

## Data Availability Statement

The RNA-sequencing datasets for the ER stress studies can be found in the NCBI Sequence Reads Archive #SRP129889 at this location https://www.ncbi.nlm.nih.gov/sra/SRP129889. Any raw data, including the OSM RNA-sequencing datasets, supporting the conclusions of this manuscript will be made available by the authors, without undue reservation, to any qualified researcher.

## Ethics Statement

The animal study was reviewed and approved by the West Virginia University.

## Author Contributions

SS and GM contributed to writing the manuscript, designing, and performing the experiments.

## Conflict of Interest

The authors declare that the research was conducted in the absence of any commercial or financial relationships that could be construed as a potential conflict of interest.
