# Supplementary material for: Janus Kinase 1 Is Required for Transcriptional Reprograming of Murine Astrocytes in Response to Endoplasmic Reticulum Stress
Source: Front Cell Neurosci. 2019 Oct 11;13:446. doi: 10.3389/fncel.2019.00446 (PMC6797841; doi:10.3389/fncel.2019.00446)
Supplement: Supplementary file 1 [file presentation_1.pptx]

## Slide 1
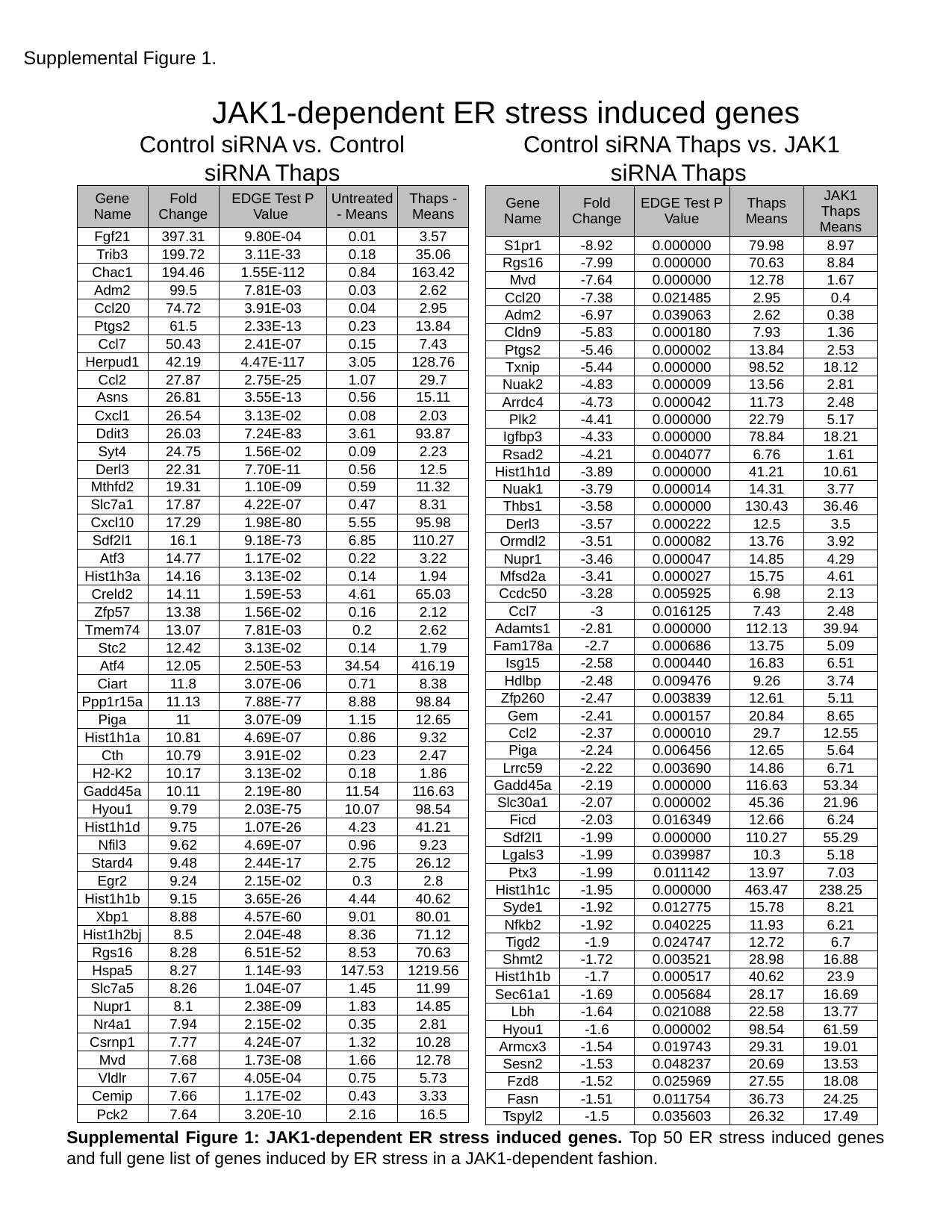

Supplemental Figure 1.
JAK1-dependent ER stress induced genes
Control siRNA vs. Control siRNA Thaps
Control siRNA Thaps vs. JAK1 siRNA Thaps
| Gene Name | Fold Change | EDGE Test P Value | Thaps Means | JAK1 Thaps Means |
| --- | --- | --- | --- | --- |
| S1pr1 | -8.92 | 0.000000 | 79.98 | 8.97 |
| Rgs16 | -7.99 | 0.000000 | 70.63 | 8.84 |
| Mvd | -7.64 | 0.000000 | 12.78 | 1.67 |
| Ccl20 | -7.38 | 0.021485 | 2.95 | 0.4 |
| Adm2 | -6.97 | 0.039063 | 2.62 | 0.38 |
| Cldn9 | -5.83 | 0.000180 | 7.93 | 1.36 |
| Ptgs2 | -5.46 | 0.000002 | 13.84 | 2.53 |
| Txnip | -5.44 | 0.000000 | 98.52 | 18.12 |
| Nuak2 | -4.83 | 0.000009 | 13.56 | 2.81 |
| Arrdc4 | -4.73 | 0.000042 | 11.73 | 2.48 |
| Plk2 | -4.41 | 0.000000 | 22.79 | 5.17 |
| Igfbp3 | -4.33 | 0.000000 | 78.84 | 18.21 |
| Rsad2 | -4.21 | 0.004077 | 6.76 | 1.61 |
| Hist1h1d | -3.89 | 0.000000 | 41.21 | 10.61 |
| Nuak1 | -3.79 | 0.000014 | 14.31 | 3.77 |
| Thbs1 | -3.58 | 0.000000 | 130.43 | 36.46 |
| Derl3 | -3.57 | 0.000222 | 12.5 | 3.5 |
| Ormdl2 | -3.51 | 0.000082 | 13.76 | 3.92 |
| Nupr1 | -3.46 | 0.000047 | 14.85 | 4.29 |
| Mfsd2a | -3.41 | 0.000027 | 15.75 | 4.61 |
| Ccdc50 | -3.28 | 0.005925 | 6.98 | 2.13 |
| Ccl7 | -3 | 0.016125 | 7.43 | 2.48 |
| Adamts1 | -2.81 | 0.000000 | 112.13 | 39.94 |
| Fam178a | -2.7 | 0.000686 | 13.75 | 5.09 |
| Isg15 | -2.58 | 0.000440 | 16.83 | 6.51 |
| Hdlbp | -2.48 | 0.009476 | 9.26 | 3.74 |
| Zfp260 | -2.47 | 0.003839 | 12.61 | 5.11 |
| Gem | -2.41 | 0.000157 | 20.84 | 8.65 |
| Ccl2 | -2.37 | 0.000010 | 29.7 | 12.55 |
| Piga | -2.24 | 0.006456 | 12.65 | 5.64 |
| Lrrc59 | -2.22 | 0.003690 | 14.86 | 6.71 |
| Gadd45a | -2.19 | 0.000000 | 116.63 | 53.34 |
| Slc30a1 | -2.07 | 0.000002 | 45.36 | 21.96 |
| Ficd | -2.03 | 0.016349 | 12.66 | 6.24 |
| Sdf2l1 | -1.99 | 0.000000 | 110.27 | 55.29 |
| Lgals3 | -1.99 | 0.039987 | 10.3 | 5.18 |
| Ptx3 | -1.99 | 0.011142 | 13.97 | 7.03 |
| Hist1h1c | -1.95 | 0.000000 | 463.47 | 238.25 |
| Syde1 | -1.92 | 0.012775 | 15.78 | 8.21 |
| Nfkb2 | -1.92 | 0.040225 | 11.93 | 6.21 |
| Tigd2 | -1.9 | 0.024747 | 12.72 | 6.7 |
| Shmt2 | -1.72 | 0.003521 | 28.98 | 16.88 |
| Hist1h1b | -1.7 | 0.000517 | 40.62 | 23.9 |
| Sec61a1 | -1.69 | 0.005684 | 28.17 | 16.69 |
| Lbh | -1.64 | 0.021088 | 22.58 | 13.77 |
| Hyou1 | -1.6 | 0.000002 | 98.54 | 61.59 |
| Armcx3 | -1.54 | 0.019743 | 29.31 | 19.01 |
| Sesn2 | -1.53 | 0.048237 | 20.69 | 13.53 |
| Fzd8 | -1.52 | 0.025969 | 27.55 | 18.08 |
| Fasn | -1.51 | 0.011754 | 36.73 | 24.25 |
| Tspyl2 | -1.5 | 0.035603 | 26.32 | 17.49 |
| Gene Name | Fold Change | EDGE Test P Value | Untreated - Means | Thaps - Means |
| --- | --- | --- | --- | --- |
| Fgf21 | 397.31 | 9.80E-04 | 0.01 | 3.57 |
| Trib3 | 199.72 | 3.11E-33 | 0.18 | 35.06 |
| Chac1 | 194.46 | 1.55E-112 | 0.84 | 163.42 |
| Adm2 | 99.5 | 7.81E-03 | 0.03 | 2.62 |
| Ccl20 | 74.72 | 3.91E-03 | 0.04 | 2.95 |
| Ptgs2 | 61.5 | 2.33E-13 | 0.23 | 13.84 |
| Ccl7 | 50.43 | 2.41E-07 | 0.15 | 7.43 |
| Herpud1 | 42.19 | 4.47E-117 | 3.05 | 128.76 |
| Ccl2 | 27.87 | 2.75E-25 | 1.07 | 29.7 |
| Asns | 26.81 | 3.55E-13 | 0.56 | 15.11 |
| Cxcl1 | 26.54 | 3.13E-02 | 0.08 | 2.03 |
| Ddit3 | 26.03 | 7.24E-83 | 3.61 | 93.87 |
| Syt4 | 24.75 | 1.56E-02 | 0.09 | 2.23 |
| Derl3 | 22.31 | 7.70E-11 | 0.56 | 12.5 |
| Mthfd2 | 19.31 | 1.10E-09 | 0.59 | 11.32 |
| Slc7a1 | 17.87 | 4.22E-07 | 0.47 | 8.31 |
| Cxcl10 | 17.29 | 1.98E-80 | 5.55 | 95.98 |
| Sdf2l1 | 16.1 | 9.18E-73 | 6.85 | 110.27 |
| Atf3 | 14.77 | 1.17E-02 | 0.22 | 3.22 |
| Hist1h3a | 14.16 | 3.13E-02 | 0.14 | 1.94 |
| Creld2 | 14.11 | 1.59E-53 | 4.61 | 65.03 |
| Zfp57 | 13.38 | 1.56E-02 | 0.16 | 2.12 |
| Tmem74 | 13.07 | 7.81E-03 | 0.2 | 2.62 |
| Stc2 | 12.42 | 3.13E-02 | 0.14 | 1.79 |
| Atf4 | 12.05 | 2.50E-53 | 34.54 | 416.19 |
| Ciart | 11.8 | 3.07E-06 | 0.71 | 8.38 |
| Ppp1r15a | 11.13 | 7.88E-77 | 8.88 | 98.84 |
| Piga | 11 | 3.07E-09 | 1.15 | 12.65 |
| Hist1h1a | 10.81 | 4.69E-07 | 0.86 | 9.32 |
| Cth | 10.79 | 3.91E-02 | 0.23 | 2.47 |
| H2-K2 | 10.17 | 3.13E-02 | 0.18 | 1.86 |
| Gadd45a | 10.11 | 2.19E-80 | 11.54 | 116.63 |
| Hyou1 | 9.79 | 2.03E-75 | 10.07 | 98.54 |
| Hist1h1d | 9.75 | 1.07E-26 | 4.23 | 41.21 |
| Nfil3 | 9.62 | 4.69E-07 | 0.96 | 9.23 |
| Stard4 | 9.48 | 2.44E-17 | 2.75 | 26.12 |
| Egr2 | 9.24 | 2.15E-02 | 0.3 | 2.8 |
| Hist1h1b | 9.15 | 3.65E-26 | 4.44 | 40.62 |
| Xbp1 | 8.88 | 4.57E-60 | 9.01 | 80.01 |
| Hist1h2bj | 8.5 | 2.04E-48 | 8.36 | 71.12 |
| Rgs16 | 8.28 | 6.51E-52 | 8.53 | 70.63 |
| Hspa5 | 8.27 | 1.14E-93 | 147.53 | 1219.56 |
| Slc7a5 | 8.26 | 1.04E-07 | 1.45 | 11.99 |
| Nupr1 | 8.1 | 2.38E-09 | 1.83 | 14.85 |
| Nr4a1 | 7.94 | 2.15E-02 | 0.35 | 2.81 |
| Csrnp1 | 7.77 | 4.24E-07 | 1.32 | 10.28 |
| Mvd | 7.68 | 1.73E-08 | 1.66 | 12.78 |
| Vldlr | 7.67 | 4.05E-04 | 0.75 | 5.73 |
| Cemip | 7.66 | 1.17E-02 | 0.43 | 3.33 |
| Pck2 | 7.64 | 3.20E-10 | 2.16 | 16.5 |
Supplemental Figure 1: JAK1-dependent ER stress induced genes. Top 50 ER stress induced genes and full gene list of genes induced by ER stress in a JAK1-dependent fashion.

## Slide 2
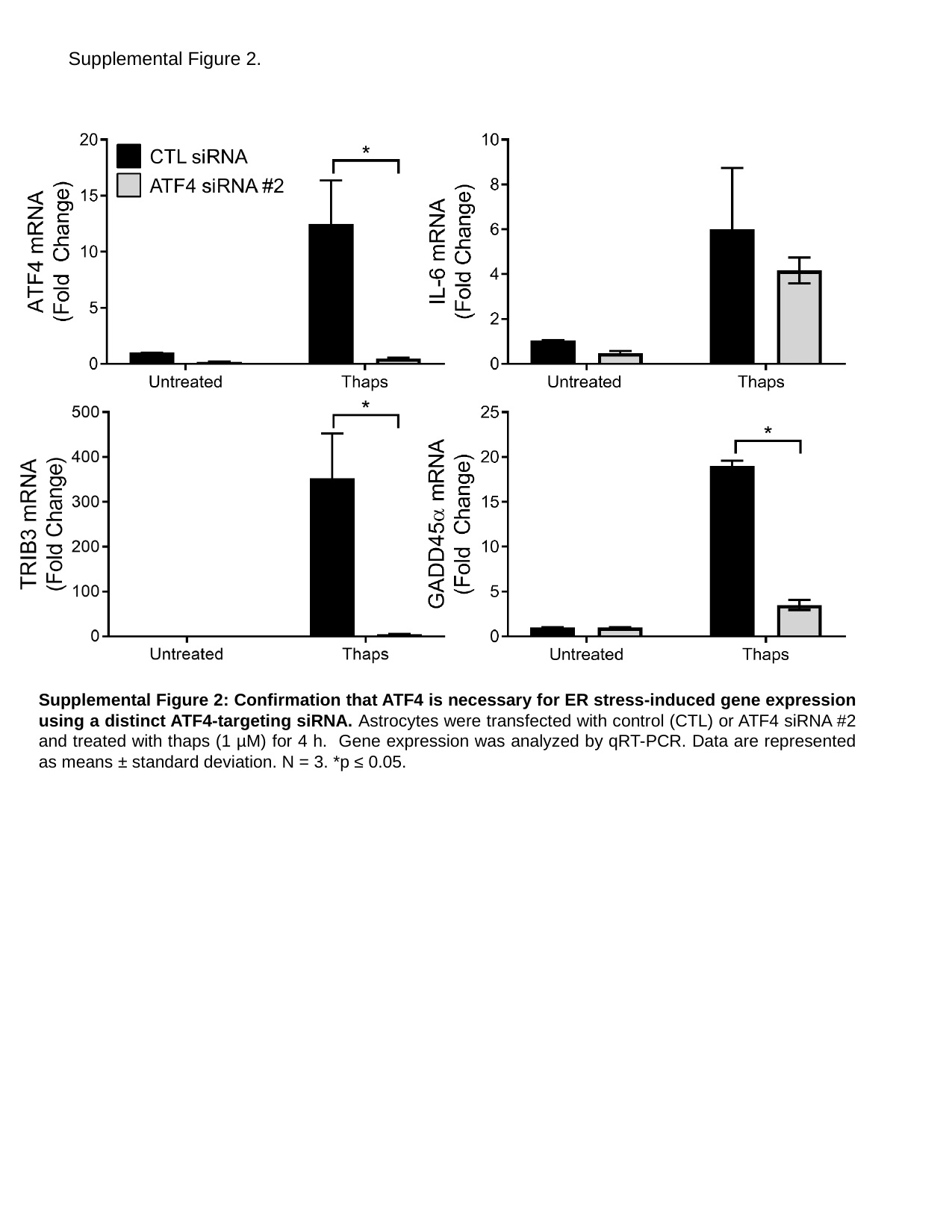

Supplemental Figure 2.
Supplemental Figure 2: Confirmation that ATF4 is necessary for ER stress-induced gene expression using a distinct ATF4-targeting siRNA. Astrocytes were transfected with control (CTL) or ATF4 siRNA #2 and treated with thaps (1 µM) for 4 h. Gene expression was analyzed by qRT-PCR. Data are represented as means ± standard deviation. N = 3. *p ≤ 0.05.

## Slide 3
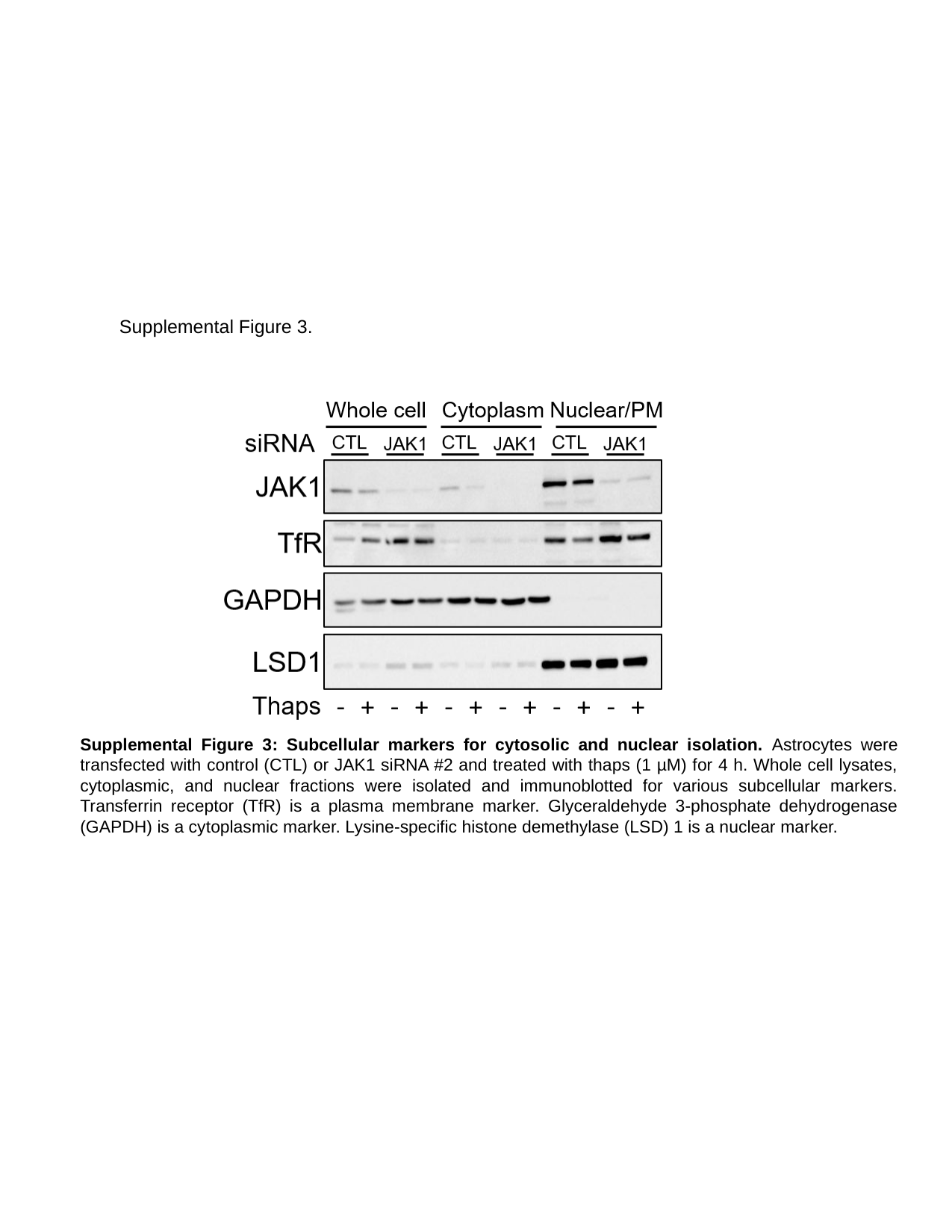

Supplemental Figure 3.
Supplemental Figure 3: Subcellular markers for cytosolic and nuclear isolation. Astrocytes were transfected with control (CTL) or JAK1 siRNA #2 and treated with thaps (1 µM) for 4 h. Whole cell lysates, cytoplasmic, and nuclear fractions were isolated and immunoblotted for various subcellular markers. Transferrin receptor (TfR) is a plasma membrane marker. Glyceraldehyde 3-phosphate dehydrogenase (GAPDH) is a cytoplasmic marker. Lysine-specific histone demethylase (LSD) 1 is a nuclear marker.
